# Use of wastewater alum-coagulation sludge as a phosphorus fertiliser – a mini review

**DOI:** 10.1007/s11356-024-32497-6

**Published:** 2024-02-17

**Authors:** Paripurnanda Loganathan, Jaya Kandasamy, Harsha Ratnaweera, Saravanamuthu Vigneswaran

**Affiliations:** 1 Faculty of Engineering, University of Technology Sydney (UTS), P.O. Box 123, Broadway, NSW 2127 Australia; 2 Faculty of Sciences & Technology (RealTek), Norwegian University of Life Sciences, P.O. Box 5003, NO-1432 Ås, Norway

**Keywords:** Aluminium sludge, Coagulation, Phosphate fertilizer, Aluminium toxicity, Soil acidity, Cationic polymer

## Abstract

The use of aluminium (Al) salts, particularly alum, in coagulation is a widespread and conventional treatment method for eliminating pollutants, including phosphorus (P) which can cause eutrophication, from wastewater. However, a significant challenge of this process is the substantial amount of sludge generated, necessitating proper disposal. Historically, land disposal has been a common practice, but it poses potential issues for plant life on these lands. Despite the associated drawbacks, sludge contains elevated concentrations of vital plant nutrients like P and nitrogen, presenting an opportunity for beneficial use in agriculture. Given the imminent scarcity of P fertilizers due to the eventual depletion of high-grade P ores, this review explores the potential advantages and challenges of utilizing Al sludge as a P source for plants and proposes measures for its beneficial application. One primary concern with land application of Al sludge is its high levels of soluble Al, known to be toxic to plants, particularly in acidic soils. Another issue arises from the elevated Al concentration is P fixation and subsequently reducing P uptake by plants. To address these issues, soil treatment options such as lime, gypsum, and organic matter can be employed. Additionally, modifying the coagulation process by substituting part of the Al salts with cationic organic polymers proves effective in reducing the Al content of the sludge. The gradual release of P from sludge into the soil over time proves beneficial for plants with extended growth periods.

## Introduction

Phosphorus (P) discharged into natural water bodies, such as lakes and rivers, through wastewater can trigger nutrient enrichment, fostering excessive growth of algae and aquatic plants and ultimately causing eutrophication (Luo et al. 2023; Muisa et al. 2020; Owodunni et al. 2023). Effectively removing and economically recovering this P for use as fertilizers not only helps control water pollution but also offers a partial solution to the anticipated scarcity of P fertilizers in the future. This scarcity is a looming concern as natural phosphate rock reserves deplete, and alarming reports suggest that high-grade P ores, vital for P fertilizer production to meet the demands of expanding food production, may be exhausted within the next 100 years (Cordell et al. 2009; Cordell and White 2014). The remaining P ore reserves are expected to have lower total or soluble P concentrations, increased impurities (such as clay), greater difficulty in physical accessibility, higher waste generation per tonne of extracted P, and consequently, elevated input and extraction costs (Cordell and White 2014). Rameshkumar et al. (2020) express concerns that the rate of P extraction far exceeds regeneration, estimating its depletion within the next 40–100 years.

Recovering P from P-containing wastewater is crucial to establish an alternative source that can contribute to alleviating the global scarcity of P. To exemplify this, let's consider a P recovery scenario from Australia, as outlined by Nur et al. (2016). In Sydney, Australia, the daily volume of wastewater exceeds 1200 ML. Assuming a standard raw sewage P concentration of 11 mg/L, Sydney's wastewater system could potentially generate 13.2 tonnes of P per day or 4,820 tonnes of P annually.

Numerous studies have focused on eliminating P from wastewater to counteract eutrophication in natural water bodies. However, only a limited subset of these studies has delved into the recovery of P for utilization as fertilizers (Zheng et al. 2023). Recently, Zheng et al. (2023) conducted a comprehensive review of prevalent water treatment methods employed for P removal from wastewaters, encompassing coagulation, biological treatment, adsorption, and membrane processes. Within these methodologies, P is concentrated in either liquid or solid waste streams, offering potential sources for P recovery. In the case of adsorption processes, P can be extracted from spent adsorbents using regeneration solutions. Membrane processes facilitate the concentration of P in wastewater into highly concentrated liquids, which can then be reclaimed into P products. The saturated filter media from spent adsorbents can also be safely applied to cropland as a fertilizer substitute. In a recent review by Arenas-Montano et al. (2021) on the utilization of spent adsorbents derived from filter adsorbents for P removal in wastewater as P fertilizers for plants, it was concluded that the efficacy of the spent adsorbent as a P fertilizer hinges on the appropriate combination of water, soil, and filter medium characteristics.

Coagulation stands out as a widely employed and conventional treatment process for eliminating pollutants, including P, from wastewater (Nayeri and Mousavi 2022; Owodunni et al. 2023; Pajak 2023; Ramal et al. 2022; Tolofari et al. 2021; Zheng et al. 2023). Aluminium (Al) salts serve as the predominant primary coagulants in water treatment processes globally, owing to their attributes of low energy consumption, straightforward design, easy operation, high efficiency, and cost-effectiveness (Dassanayake et al. 2015; Muisa et al. 2020; Pajak 2023; Ramal et al. 2022; Tolofari et al. 2021; Zheng et al. 2023). Coagulation utilizing Al salts efficiently eliminates P from wastewater, transforming it into a valuable resource for fertilizer production and thereby mitigating a significant environmental concern (Manamperuma and Ratnaweera 2015). This paradigm shift implies that wastewater sludge generated from the coagulation process is no longer treated merely as waste but is instead recognized as a valuable resource.

Tony (2022) compiled data summarizing the annual production of Al sludge in the USA, UK, Europe, and Asia over the past two decades (1996–2019), revealing a range between 0.1 and 2.3 million tonnes. Zhao et al. (2021a) noted challenges in obtaining accurate global data on Al sludge production but reported that China produces the highest quantity globally at 2.3 million tonnes per year. They reported that the amount of Al sludge would continue to increase worldwide due to the increasing demand for clean water with the rapid escalation of world population and urban expansion. Okuda et al. (2014) suggested a global daily estimate of 10,000 tonnes of Al sludge production. In many countries, these substantial volumes of sludge are commonly disposed of in landfills or discharged directly into rivers, posing environmental risks (Muisa et al. 2020; Nayeri and Mousavi 2022). These practices involve high disposal and transportation costs, legal restrictions, necessitating an urgent need for effective sludge management. In some places disposal in the environment was prohibited because of the risk of contaminants in the sludge (Nunes et al. 2021; Nayeri and Mousavi 2022). The application of Al sludge to agricultural lands as a P fertilizer not only resolves the disposal issue but also serves a beneficial purpose. However, this practice may have adverse effects, such as Al toxicity to plants. Additionally, despite Al salts removing P from wastewaters and generating sludge with significant P content suitable for fertilizers, the availability of P to plants may be limited. This is due to the strong binding of P to Al in the sludge (Rigby et al. 2013; Toor et al. 2019) or the chemical precipitation of P by Al in the sludge (Johannessen et al. 2012; Øgaard and Brod 2016). Furthermore, the Al hydroxide precipitate in the sludge strongly adsorbs native P in the soil, leading to fixation and making it less available to plants (Johannessen et al. 2012; Kim et al. 2002; Loganathan et al. 2014; Toor et al. 2019). Even if these constraints are addressed through chemical means, the global P production from Al sludges remains a small fraction of the annual mined P (20 million tonnes) (Cordell and White 2014). The amount of iron sludge produced by iron salts does not significantly alter this percentage, as iron salts are less commonly used in coagulation compared to Al salts (Matei et al. 2022). Nevertheless, the sludge produced can still be a cost-effective P source for agricultural lands near treatment plants, simultaneously aiding in resolving the waste disposal challenge faced by the treatment plants.

Another concern related to the application of Al sludge on agricultural lands involves the potential presence of pollutants in the sludge, including heavy metals (Cd, Cr, Hg, Pb, Zn, Cu), toxic organic compounds, and pathogenic organisms, which can pose human health risks through the food chain. Studies indicate that the risks are likely lower when applying Al sludge to soils compared to sewage sludges, which generally exhibit higher concentrations of these contaminants (Nunes et al. 2021). Dassanayake et al. (2015) and Zhao et al. (2021b) conducted reviews on the concentrations of these contaminants in Al sludges from various regions, concluding that Al sludge poses minimal environmental hazards compared to sewage sludge from wastewater treatment plants. The levels of heavy metals in Al sludge reported in the literature were found to be significantly below the regulatory levels set by the US Environmental Protection Agency (US-EPA) (Dassanayake et al. 2015). Zhao et al. (2021b) reported concentrations of Pb, Cr, Cd, and Hg in different parts of the world as 22 ± 12, 20 ± 7, 0.12 ± 0.02, and 0.46 ± 0.11 mg/kg, respectively. Pajak (2023) review reported values of 2–37, 14–81, 2–1600, and 0.02–0.66 mg/kg, respectively, for these metals. However, it's essential to consider that the feedwater used in the coagulation process may partly consist of industrial wastewaters with elevated concentrations of some heavy metals and organic compounds. This could lead to increased concentrations of pollutants in the resulting Al sludge. Therefore, it is crucial to assess the quality of the sludge for these contaminants before applying it to food crops. This precautionary measure ensures the safe utilization of Al sludge in agriculture without compromising food safety or environmental integrity.

The objectives of this review paper were threefold: (1) To conduct a comparative analysis of the total and soluble P contents in Al sludges versus commercial P fertilisers. (2) To assess and compare the plant-availability of P in Al sludges with that of commercial P fertilizers across soils with diverse properties. (3) To propose effective methods for addressing constraints associated with the utilization of Al sludge on agricultural lands. While existing literature includes comprehensive reviews on the use of P recovered through adsorption (Luo et al. 2023; Zheng et al. 2023) and membrane processes (Li et al. 2021; Rameshkumar et al. 2020), there is currently a notable absence of a comprehensive review specifically addressing P recovery by coagulation using Al salts for use as fertilizer. This paper aims to bridge this knowledge gap by presenting a review that sheds light on the beneficial utilization of substantial quantities of Al-sludge generated in water treatment plants globally. Methods to overcome constraints linked to the utilization of Al sludge on agricultural lands such as Al toxicity to plants and potential for Al in the sludge to induce fixation of the available P in the soil were presented.

## Materials and methods

The review was systematically conducted through an extensive literature search, utilizing prominent scholarly databases such as Google Scholar®, Web of Science, and Scopus. Articles primarily from the period spanning 2000 to 2023, with a focus on the later years, were collected, thoroughly analysed, and pertinent information was integrated into the review paper. Keywords employed for the search encompassed topics such as the global application of Al and iron salts for coagulation in water treatment, worldwide production volumes of Al sludge, P content and plant availability in Al sludges, trends in P fertilizer production globally, chemical reactions of Al sludge in soils, and the agricultural land application of Al sludge. In an effort to access recently published review articles, the keyword 'Review' was included in the search criteria. Additionally, a search was conducted for recent review articles addressing P removal and recovery as P fertilizer from wastewater, particularly focusing on adsorption and membrane technology. Notably, it was observed that while numerous reviews exist for these technologies, there is a scarcity of reviews pertaining to coagulation using Al salts. The selection of review articles primarily from the last three years ensured that the analysis presented in this review remains current and reflective of recent advancements in the field.

In contrast to Al sludges generated in wastewater treatment plants, those originating from drinking water treatment exhibit significantly lower P content, ranging from 0.008% to 0.16%, primarily due to the inherently low concentration of P in the feed water (Jonasson 1996; Truong and Kim 2021; Lombi et al. 2010). The exceedingly low P concentrations in sludges from drinking water treatment processes impede their suitability for use as P fertiliser, even when applied in higher quantities. As a result, sludges from drinking water treatment are excluded from consideration in this review.

## Sludge P content and solubility

The wet Al sludge produced in the treatment plant is voluminous and gelatinous, typically containing over 98% water (Nayeri and Mousavi 2022) with solid contents ranging from 1 to 5% (Jonasson 1996; Tony 2022). The P content of the sludge, post-drying through various methods, exhibits significant variability due to differences in P concentrations in wastewater and drying techniques (Table 1). The P contents are notably lower than those found in commercial fertilizers. For instance, a widely used commercial P fertilizer, diammonium phosphate (DAP), boasts a P content of 15% (Loganathan et al. 2003) (Table 1). As reported in the literature, the P content of many sludges falls within the range of 1% to 5.6% P by dry weight mass (equivalent to 7%-37% of DAP) (Table 1). Consequently, larger quantities of sludge are required to achieve equivalent plant P uptake as conventional fertilizers. An additional challenge with the P in sludge is its predominantly insoluble nature, making it less readily available for plant uptake compared to organic fertilizers (Table 1). Nevertheless, when wastewater Al sludge is applied to soils, it not only serves as a P source but also offers other essential nutrients for plant growth, including nitrogen (N), similar to organic fertilizers (Table 1). The N content of wastewater Al sludge is comparable to that of many organic N fertilizers (Bergstrand 2022). Kim et al. (2002) observed increased plant growth and root elongation in an acid soil pot trial when Al sludge was applied, attributing it in part to the higher N content (0.99%) in the sludge, which exceeded the N content in the native soil used in the trial (0.07%).

**Table 1:** Total P content and P fraction concentrations in wastewater alum sludges, mineral fertilizer and organic manure

Sludge source	Total Al (%)	Total P (%)	Total N (%)	Plant-avail. P (mg/kg)	Water-sol- P (mg/kg)	Organic P (mg/kg)	Al-bound P (mg/kg)	Reference
Al sulphate (thermal hydrolysis 160–165 °C), Norway	4.34	3.1	NA	NA	26.5	3039	11850	Krogstad et al. (2005)
Al agent (PAX 21), pasteurisation (65–70 ◦C), Norway	7.17	2.3	NA	NA	6.5	2475	7400	Krogstad et al. (2005)
Al sulphate, Norway (dried 40 °C)	8.4	3.1	5.2	21.2*	NA	2500	NA	Øgaard and Brod (2016)
Poly Al chloride sludge, filtered and dried for 48 h at 105 °C. Ground < 0.5 mm. Korea	16.3	5.6	NA	2100	22.4	NA	16240	Toor et al. (2019)
Alum sludge, dewatered (18% soils) Manitoba, Canada	1.1	1.2	NA	808**	NA	NA	NA	Tolofari et al. (2021)
Alum sludge, dewatered/stock piled-1-week in field, Australia	7.5	3.7	5	NA	NA	NA	NA	Rigby et al. (2013)
Al sulphate sludge, dried 100 °C, California, USA	NA	1.7	2.7	NA	NA	NA	NA	Gestring and Jarrell (1982)
DAP (fertilizer)	Nil	15	18	100	100	Nil	Nil	McLaren and Cameron (1996)
Cow manure	Nil	0.2	0.7	NA	Nil	⁓2000	Nil	McLaren and Cameron (1996)

^*^ CaCl_2_ extractable P, **bicarbonate extractable P, NA -not available

The various forms of P in sludge are typically determined through P fractionation using a sequential extraction method. This technique categorizes P into Al-P, Fe–P, Ca-P, and reductant soluble-P (Hedley et al. 1982; Rivaie et al. 2008). Sequential chemical extraction of sludges has consistently revealed that NaOH-extractable inorganic P, specifically defined as Al-/Fe-bound P (fixed P fraction), tends to be the predominant P fraction (Øgaard and Brod 2016). In contrast, the labile P fraction (considered the plant-available fraction), extracted by 0.5 M NaHCO_3_, consistently appears as the lowest P fraction (Øgaard and Brod 2016). However, sludge also contains a notable concentration of organic P (approximately 0.3%, as indicated in Table 1), dependent on the wastewater source. In this organic form, additional P can become available to plants in a relatively short period of 50 days through the mineralization of organic P (Tolofari et al. 2021).

Cox et al. (1997) observed that the loosely bound P fraction, representing plant-available soil P, in a highly acidic soil (pH 4.4) decreased with increasing rates of Al sludge application. Simultaneously, the soil fractions of Ca-P, Al-P, and Fe–P increased with sludge application rates, suggesting an elevated fixation of P to calcium, aluminum, and iron compounds in the soil. In a similar vein, Jonasson (1996) documented a reduction in plant-available soil P fraction during laboratory incubation of two acid soils with Al sludge. This reduction coincided with an augmentation in Ca-, Al-P/Fe–P (NaOH extractable inorganic P), and Organic P (NaOH extractable organic P).

Krogstad (2010) indicated that the plant availability of P from sludge generated in wastewater treatment plants (WWTP) using Al coagulants was approximately 10%, in contrast to sludge obtained from the biological treatment process at WWTPs and mineral fertilizers. In a previous study, Krogstad et al. (2005) compared sludges from five different coagulation/flocculation treatment plants and noted that the treatment history of sewage sludge influenced the availability of sludge P to plants. They found that sludges produced through biological treatment processes exhibited a P fertilizing ability comparable to inorganic fertilizers, while P precipitated using iron and Al chemicals resulted in sludges with very low P fertilization value. Sequential chemical extraction of sludges revealed that NaOH-extractable P, considered to be Al-/Fe-bound P (fixed P fraction), was often the most substantial P fraction (Øgaard and Brod 2016). Meanwhile, the labile P fraction (plant-available fraction), extracted by 0.5 M NaHCO_3_, was typically the lowest fraction (Øgaard and Brod 2016).

## Plant availability of P in sludge-amended soils

The assessment of sludge as a P fertilizer for plants necessitates an examination of its impact on soil reactions and the subsequent uptake of P by plants. Various factors, such as soil composition, mineral content (especially Al oxide/hydroxide), pH, and organic matter content, can influence the availability of P in sludge to plants, as outlined in Table 2. Øgaard and Brod (2016) conducted a study on the fertilizing efficacy of multiple sludges containing P, derived from wastewater treatment plants in Norway, where wastewater underwent coagulation with Al or iron (Fe). These sludges underwent diverse treatments, including liming before being applied to soils. Their findings revealed that the availability of P to ryegrass increased over the course of plant growth, albeit remaining lower than that of artificial fertilizer. The limed sludges exhibited the highest concentration of labile soil P, constituting 17% and 38% of the total P. In contrast, the other nine sludges contained ≤ 5% of the total soil P. This disparity was attributed to lime application, which transformed less available Al phosphate (AlP) or iron phosphate (FeP) into more accessible calcium phosphate (CaP). The calculation of the mineral fertilizer equivalent, based on ryegrass P concentration, indicated that limed sludges had the highest values. Additionally, the limed sludges exhibited the highest soil pH, surpassing 8.

**Table 2:** Effect of soil application of Al sludge on P availability to plants

Experimental treatments	Soil properties	Performance	Reference
Al sludge vs commercial fertilizer (4 rates), Switchgrass, pot experiment, 3 harvests, 50 d interval. P rates based on Olsen’s available P in sludge	pH 8.3, low avail P (3 mg/g), organic C (25 g/kg)	1st harvest had no difference between sludge and fertilizer. Later harvests had P uptake higher for sludge. Sludge has org P which released P gradually. Soil available P decreased with time for commercial fertilizer due to soil P fixation	Tolofari et al. (2021)
Same as above except the crop was maize, 4 harvest, 45 d interval	Same as above	Same results as above except sludge was better even for the first harvest	Tolofari et al. (2022)
Al/Ca precipitated sludge (40 kg P/ha) compared with mineral fertilizer (5 rates, 15–60 kg/ha). Field expt. in Ireland for 1 year with rye grass	pH 5.6, organic C 1.8%, Morgan’s available P 2.86 mg/L (P deficient soil)	Mineral fertilizer had higher plant P uptake at 1st harvest (P highly soluble initially). Fourth harvest sludge produced higher P uptake. Slow availability of P from sludge. Sludge P less prone to lock-up in soil than mineral fertilizer P	Ashekuzzaman et al. (2021)
11 types of sludges compared in a pot trial on ryegrass. 1st cut at 6 weeks, later 4 weeks apart. Sludges 225 mg P/pot, Mineral P fertilizer 3 rates (75–225 mg P/pot)	pH 7.2, organic C 0.26%, Available P 0.47 mg/kg	Plant-available P lower than that of mineral fertilizer. Plant availability more for Fe sludge than Al sludge because of lower solubility of Al sludge. Liming the sludge increased P availability because of shift from Al/Fe P to CaP^−^ still lower P availability than mineral fertilizer	Øgaard and Brod (2016)
DAP fertiliser (20 kg P/ha) vs Al sludge, 5 rates (124–744 kg P/ha). 2 yr-field trial in Western Australia on barley and wheat	pH 5, organic C 0.74%, Available P (Olsen’s) 7 mg/g	Plant P concentration increased with increased rate of sludge but lower than that for DAP in both years for both crops. Sludge had less P availability than DAP even when applied at higher rates because P was bound to Al	Rigby et al. (2013)
Al sludge applied to two soils at 6 rates (equivalent to 5–500 tonnes/ha). Pot expt. for 4 weeks on lettuce	Sandy soil pH 7.1, Clay soil pH 5.5	P uptake declined with increase alum rate. Plant growth also declined, more with high pH soil due to sludge induced P deficiency. Not due to Al toxicity. pH slightly declined in sandy soil but increased in clay soil	Lombi et al. (2010)
One of the 5 sludges tested in pot trial (73 days) with ryegrass was Al based. Inorganic fertilizer 30–120 kg/ha; Sludge 20 tonnes/ha	pH soils 5.7, 6.74; Organic C (%): 5.61, 5.28	Al sludge produced low P fertility value in this short-term experiment. Sludge from biological treatment produced highest P fertility comparable to inorganic fertilizer. Total P will keep accumulating in soil from Al sludge over long term use	Krogstad et al. (2005)
Al sludge after treatment of alum and lime tested in a 5- week pot trial. 4 rates 1.5–18% moist sludge	pH 4.0 (very acidic), available P 0.76 mg/kg, Organic matter 4.5%	Reduced P uptake due to P adsorption by the increased amounts of alum with Al and Ca. Sludge increased root elongation, soil acidity buffering capacity, hydraulic conductivity, water holding capacity	Kim et al. (2002)

Tolofari et al. (2021) conducted a pot trial to assess the availability of P in alum sludge compared to a commercial fertilizer (monoammonium phosphate, MAP) using switchgrass grown in soil with low P availability (plant-available bicarbonate-P, 3 mg/kg) and high pH (8.3). Five P application rates were tested, and plants were harvested thrice at 50-day intervals (3 cycles). P application was determined based on the bicarbonate-extractable P concentration of the sludge (plant-available fraction measured by soil test, Olsen-P) rather than total P. This approach facilitated the comparison of treatments between sludge and MAP since a significant portion of the total P in the sludge was not expected to be readily available for plant uptake during a short growth cycle. Results revealed no significant difference in P uptake between alum-P sludge and MAP during the first cycle. However, in cycles 2 and 3, alum-P sludge exhibited significantly greater P uptake compared to MAP. While P uptake from MAP remained relatively consistent across the three cycles, there was a slight decrease with increasing cycles. Conversely, plants fertilized with alum-P sludge demonstrated an average increase in P uptake in Cycle 3 compared to Cycle 1. The decreased P uptake in the MAP treatment was attributed to the binding of soluble inorganic P to soil metal compounds of Ca, Mg, and Al. The observed increase in P uptake from sludge suggested a gradual release of P from the organic source in the sludge (NaOH-Po fraction) over time through the mineralization process. Additionally, the abundant organic nitrogen in the sludge (Table 1) likely underwent mineralization, supplying nitrate and ammonium to plants, thereby promoting growth. The authors concluded that switchgrass could access more P from alum-P sludge than MAP, utilizing its root system, as P was gradually released from alum-P sludge over the cumulative cyclical growth period, evident in the gradual increase in P uptake in each cycle. This led to the overall assessment that alum-P sludge serves as an effective source of available P for growing perennial crops, specifically switchgrass, in soils characterized by high pH and low plant-available P. Notably, at higher soil pH levels, P fixation/precipitation with Al is lower than at lower pH (Loganathan et al. 2014).

In contrast to the alkaline soil study conducted by Tolofari et al. (2021), a 5-week pot trial using Indian mustard plants in a highly acidic soil (pH 4.0) revealed diminished P uptake in both leaves and roots as the application of alum sludge increased. The alum sludge, sourced from a Korean water treatment plant, had a low P content (plant-available P of 0.16 mg/kg) (Kim et al. 2002). The reduced P uptake was attributed to increased P fixation in the acidic soil by the alum treatment containing large amounts of Al and Ca. However, despite the decreased P uptake, plant growth, including root elongation, showed improvement with higher sludge application rates. This enhancement in plant growth was explained by the positive impact of sludge addition on the physical properties of the soil, such as bulk density, hydraulic conductivity, and water-holding capacity. The increased root elongation was considered a contributing factor to improved root processes, including the release of root exudates, which, in turn, mobilized plant nutrients for more efficient utilization (Ma et al. 2022). Additionally, the relatively high N content in the alum sludge (0.99%) compared to the soil (0.07%) was identified as another factor influencing increased plant growth. Kim et al. (2002) concluded that while their short-term pot trial suggested that land application of alum sludge could serve as an economical disposal method, further validation through long-term field trials was necessary. They also proposed that the reduced P uptake by plants could potentially be addressed by supplementing sludge application with additional P fertilizers.

A two-year field study conducted on wheat (year 1) and barley (year 2) growing in an acid sand (CaCl_2_ pH 5.0, bicarbonate-P 7 mg/g) in Perth, Australia (Rigby et al. 2013) reported similar findings as in the pot study of Kim et al. (2002). Rigby et al. (2013) also reported that alum would have produced high concentrations of soluble Al at the low pH of the soil causing precipitation of P and hence low P availability to plants. The grain yield for the crops treated with inorganic fertilizer was 44% higher than the control in year 1 and 58% higher in year 2. However, grain yield for the crops treated with alum sludge applied at an equivalent available N rate to the inorganic fertilizer was 62% (year 1) and 69% (year 2) of the yield achieved by the inorganic fertilizer, but higher than the control treatment. Rigby et al. (2013) reported that soil analysis indicated that fertilizing with alum sludge increased the P concentration in the P deficient acid soil even though it was not readily available for crop uptake. The alum treated soil contained a greater proportion of P which was chemically bound to Al compounds in the acid soil. They concluded that although alum sludge was not a suitable source of P in acid soils with low P status, it may be a suitable amendment supplying N in certain soil types where there is sufficient P for crop growth, or where soils have a poor P adsorbing capacity.

**Fig. 1:**
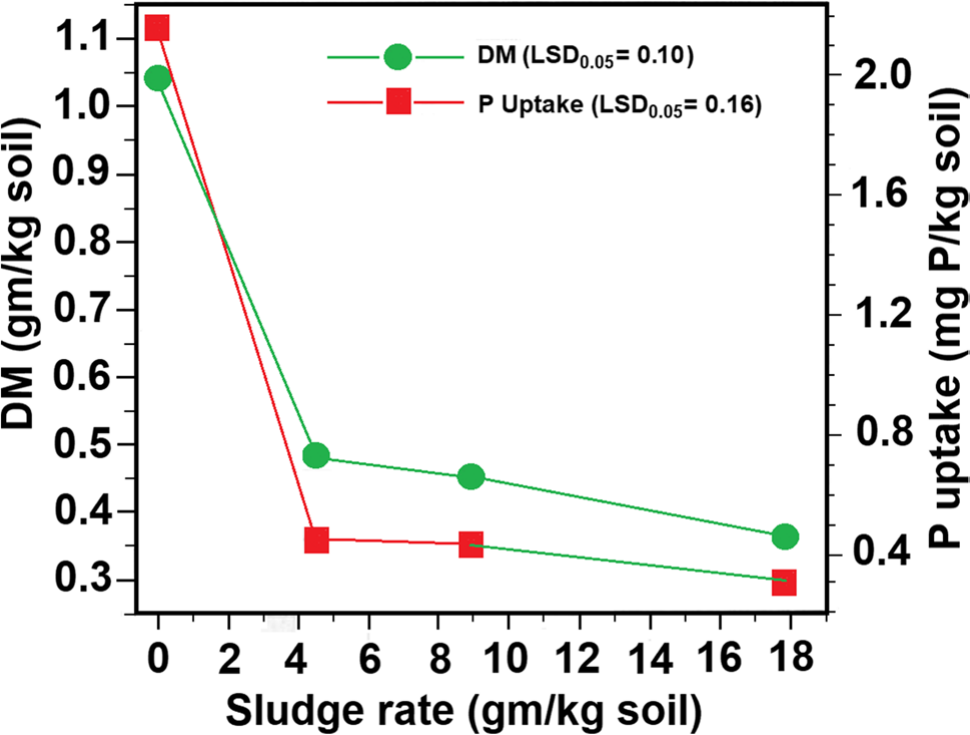
Effect of alum application on plant dry matter (DM) yield and P uptake (mg P in plant dry matter/ kg soil) of the second wheat crop in a highly acidic soil (pH 4.4) (modified from Cox et al. (1997))

Cox et al. (1997) observed that escalating the application of alum sludge (0.24% P) to a highly acidic soil (pH 4.4) with high plant-available soil P (Mehlich No 1 P of 63.2 mg/kg) resulted in a reduction in wheat uptake of P and a decrease in dry matter yield in a pot trial (Fig. 1). The decline in dry matter yield was statistically significant only at the first application rate, while for P uptake, significance was noted at both the first and third application rates. This trend was linked to a decrease in the loosely bound P fraction (plant-available P) in the soil with higher sludge application rates (Table 3).

**Table 3:** Effect of alum sludge incorporation to a highly acidic soil on soil P fractions of the entire soil volume (0–15 cm depth) after second wheat crop in a pot trial (Cox et al. (1997))

Sludge rate	Loosely bound-P (plant available soil P)	Al-P	Fe–P	Ca-P
g/kg soil	mg P/kg soil)			
0	3.5	111.8	47.6	27.7
4.45	2.4	117.0	45.4	25.5
8.9	1.7	117.3	50.7	21.3
17.8	2.0	121.2	55.8	25.5
ANOVA	*	NS^**^	*	NS**

^*^ANOVA significant at the 0.01 probability level (LSD values were not reported by Cox et al. (1997)); ^**^ NS = Not significant

## Overcoming constraints of Al sludge usage on agricultural lands

Three primary constraints linked to the utilization of Al sludge on agricultural lands include Al toxicity to plants in acidic soils, stemming from elevated concentrations of Al in the sludge; the low levels of total and plant-available P in the sludge, necessitating the application of large volumes in comparison to commercial fertilizers; and the potential for Al in the sludge to induce fixation of the already available P in the soil (native soil P).

The initial challenge posed by sludge-induced Al toxicity to plants can be mitigated by incorporating lime into the soil, especially when dealing with acidic soil, to decrease the solubility of Al in the soil. This approach is a well-established practice in the field of soil science (Haynes and Mokolobate 2001; McLaren and Cameron 1996; Nwachuku and Loganathan 1991). Alternatively, Al sludge itself can undergo treatment with lime before being applied to the soil to reduce Al solubility. According to Øgaard and Brod (2016), the solubility of P in alum increases after liming, as Al is precipitated at higher pH levels. This transformation results in the conversion of the Al-bound P fraction to a Ca-bound P fraction, which has a lower potential for P fixation (Loganathan et al. 2014).

The application of lime on the surface can effectively address acidity in the upper layer of soil but is insufficient for correcting subsoil acidity due to the limited solubility of lime, preventing its migration to the subsoil. To tackle subsoil acidity comprehensively, gypsum, which boasts greater solubility than lime, has been employed in conjunction with lime (Sumner 1993; Wang et al. 1999). To extend the incorporation of alum sludge to deeper soil depths, as demonstrated by Cox et al. (1997), it becomes imperative to apply both lime and gypsum. This combined approach not only mitigates Al toxicity throughout the entire soil profile but also facilitates deeper root growth for plants.

The mitigation of Al toxicity can also be achieved by incorporating organic matter into the soil before the application of Al sludge. The reduction in phytotoxic Al resulting from the addition of organic matter is attributed to the complexation of soluble Al species with organic molecules derived from the organic matter, coupled with adsorption on the organic material (Haynes and Mokolobate 2001). Zhao et al. (2020) observed that applying canola straw, peanut straw, and a commercial organic fertilizer to a highly acidic soil (pH 4.32) alleviated Al toxicity and increased the yield of sweet potato and canola. While the yield increase was significant only for the organic fertilizer, all treatments led to a substantial reduction in Al concentration in the soil solution.

The challenge posed by the low concentrations of total and plant-available P in sludge, requiring substantial amounts for application compared to commercial fertilizers, can be addressed by substituting a portion of the Al salts used for coagulation with organic polymer coagulants. Manamperuma and Ratnaweera (2015) demonstrated that incorporating a smaller dose of cationic polymer during the coagulation process increases the plant-available P in sludge. As the Al content in sludge originates solely from the Al salt used in coagulation, substituting part of the salt with a cationic polymer reduces the Al content, leading to decreased P fixation by Al and thereby increasing plant-available P. Both cationic polymers and positively charged Al species (Al hydrolysis species) destabilize colloidal systems during coagulation (Manamperuma and Ratnaweera 2015). The subsequent flocculation of destabilized (coagulated) colloids is primarily achieved through a polymer bridging mechanism (Loganathan et al. 2020). Despite the higher cost of polymers compared to alum, the required dose is minimal, offsetting the higher expense and offering additional advantages. For instance, Dong et al. (2014) discovered that the optimal dose of the natural polymer chitosan (costing $51/kg) for coagulating turbidity in synthetic algal turbid water was only 0.4 mg/L, compared to Al sulphate (costing $5.8/kg) with an optimal dose of 20 mg/L. This cost comparison illustrates the economic advantage of using chitosan over Al sulphate (last column of Table 4). In their coagulation experiment, Manamperuma and Ratnaweera (2015) simultaneously added Al sulphate and a cationic polymer to synthetic wastewater (pH 7.5) at various combinations (0–5 mg/L polymer, 5–15 mg/L Al) and investigated the coagulation process. They reported similar removals of suspended solids and P for various ratios of polymer and Al doses. Notably, at lower Al doses (5–7 mg/L), the impact of cationic polymers (1, 3, and 5 mg/L) was substantial, with the influence diminishing as the Al dosage increased (10–15 mg/L).

**Table 4:** Comparison of optimal dosages (OD) of coagulant/flocculants to treat one m^3^ of algal turbid water and corresponding algal turbidity removal efficiencies (TRE) using chitosan and Al sulphate (data from Dong et al. (2014))

Coagulant/flocculant	Price (US $/kg)	Optimum dose (g coagulant/ m^3^ water)	Turbidity removal efficiency (%)	Cost (US $/m^3^ water)
Chitosan	51.2	0.4	94.7	0.02
Al_2_(SO_4_)_3_	5.8	20.0	89.2	0.12

In a parallel investigation, Al sulphate (1–10 mg/L) and a cationic polymer at a significantly lower concentration (0.2–1 mg/L) were sequentially added to synthetic water containing humic acid and kaolin (Guo et al. 2015). The study reported that polymer addition exhibited higher coagulation efficiency over a broader pH range, along with an increase in floc size and its growth rate compared to the addition of Al sulphate alone. Guo et al. (2015) attributed this enhanced performance to the high cationic charge density and long chain of the polymer, which could intensify the effects of charge neutralization and adsorption bridging. It's noteworthy that this study did not measure the Al content of the sludge produced. Given that a lower polymer dose produced higher coagulation efficiency than Al sulphate in the study by Guo et al. (2015), a substantial portion of the Al sulphate could be replaced by a smaller dose of polymer. The combination could still achieve the same or even higher coagulation efficiency compared to using Al sulphate alone. This approach would align with the findings of Manamperuma and Ratnaweera (2015) and contribute to reducing the Al content of the sludge produced.

Partially substituting Al salts with polymers in the coagulation process would also mostly alleviate the third constraint mentioned in the literature, namely the fixation of native soil P by the introduced Al sludge (Kim et al. 2002; Rigby et al. 2013).

## Conclusions and recommendations

The primary issue with the Al sludge is the elevated concentration of Al, which can be mitigated by replacing a portion of the required Al salts with a reduced dose of cationic polymers in the coagulation process. These polymers offer additional advantages beyond facilitating coagulation. Moreover, polymers serve as more efficient coagulants/flocculants compared to Al salts, as their coagulation/flocculation mechanisms involve both charge neutralization and inter-particle bridging, whereas Al salts primarily operates through charge neutralization.

The impact of Al sludge on plants varies, with effects ranging from adverse to beneficial depending on factors such as soil pH, soil phosphate content, duration of plant growth, and sludge characteristics. Studies indicate that applying sludge to acid soils can hinder plant growth due to Al toxicity and a reduction in P availability caused by increased P fixation to the soil. These challenges can be mitigated by incorporating lime into the sludge to elevate its pH and by adding lime, gypsum, and organic materials to the soil to rectify surface and sub-surface soil acidity before sludge application. Additionally, research suggests that if the soil is already deficient in plant-available P, sludge application may further decrease P availability due to fixation with Al. To address this issue, supplementing sludge application with P fertilizer is recommended.

The application of sludge typically proves advantageous for crops with extended growth periods, such as perennial crops, as it gradually releases nutrients (N, P) throughout the crop's growing period. Apart from P bound to Al, iron, and calcium compounds in the sludge, substantial quantities of N and P are present in the organic fraction of the sludge. Through the gradual mineralization process facilitated by microbial biomass in the sludge, these nutrients can become available to plants.
